# Development and Evaluation of Alginate- and Carrageenan-Incorporated Scaffold for Bone Regeneration: An In Vitro Study

**DOI:** 10.7759/cureus.61139

**Published:** 2024-05-26

**Authors:** Devika Bajpai, Kaarthikeyan G

**Affiliations:** 1 Periodontics, Saveetha Dental College and Hospitals, Saveetha Institute of Medical and Technical Sciences, Saveetha University, Chennai, IND

**Keywords:** regeneration, carrageenan, alginate, scaffold, tissue engineering, periodontitis

## Abstract

Introduction: Periodontitis, a persistent inflammatory condition, results in the deterioration of both the hard and soft tissues in the periodontium, leading to the formation of intrabony defects. Restoring the lost tissues, particularly bone, is possible through tissue engineering techniques utilizing scaffolds made from different polymers. Consequently, this research focuses on creating and assessing a scaffold infused with alginate (Sigma Aldrich, Gillingham, UK) and carrageenan (Sigma Aldrich, Gillingham, UK) for the purpose of bone regeneration.

Methods: An in vitro investigation was conducted to assess the characteristics of the recently formulated scaffold. Spectroscopic analysis, tensile strength testing, scanning electron microscopy (SEM) analysis, and degradation testing were carried out to evaluate both the physical and biological attributes of the scaffold.

Results: IBM SPSS Statistics for Windows, V. 1.2 (IBM Corp., Armonk, NY, USA) was used for statistical analysis. A one-way ANOVA test was done to determine the significance of tensile strength, and a paired t-test was done to check the significance of the degradation test. The in vitro research unveiled notable distinctions in the physical and biological attributes between the scaffold infused with alginate and carrageenan and the PerioCol® (p<0.05).

Conclusion: The scaffold incorporating alginate and carrageenan demonstrated superior outcomes concerning parameters such as tensile stress and strain, degradation rate, percentage bone volume, and object surface density when contrasted with the conventional PerioCol®. Therefore, the scaffold infused with alginate and carrageenan emerges as a promising candidate for bone regeneration.

## Introduction

Periodontal disease, a persistent inflammatory condition, stems from the presence of numerous microbial organisms. It triggers an inflammatory response, resulting in the breakdown of the supportive tissues surrounding the teeth, namely, the gingiva, bone, and periodontal ligament. While nonsurgical mechanical therapy might suffice for resolving periodontal inflammation and removing subgingival microbial biofilm, the restoration and integrity of the periodontium necessitate the regeneration of alveolar bone defects [1].

Traditional surgical methods like open flap debridement offer substantial access for assessing and cleansing root surfaces; however, they cannot fully restore or reconstruct the lost structure [2]. The reconstruction of the defect involves intricate interactions among cells and molecules [3]. Regenerative techniques often incorporate bone grafts for osteogenesis. Migration of epithelium in the defect region impedes the growth of periodontal ligament fibres. To counteract this, barrier membranes like conventional collagen membrane (PerioCol® derived from fish collagen) are used to inhibit the early migration of epithelial cells in the defect. This procedure is known as guided tissue regeneration (GTR) [4].

Inadequate success in achieving regeneration through standard GTR procedures may lead to various defects in the alveolar ridge as periodontal disease progresses. Addressing these defects for subsequent augmentation can be challenging. While autogenous block grafting has been traditionally favored for such cases, it inevitably results in patient morbidity. To tackle these issues, Langer and Vacanti introduced a potential approach for regenerating lost periodontal tissues (tissue engineering) in 1993. This interdisciplinary area merges materials science with biocompatibility, incorporating cells, natural or artificial scaffolds, and signaling molecules to foster the generation of new tissues [5].

Scaffolds derived from polymers (natural or synthetic) are widely utilized in tissue engineering, as they emulate the characteristics of the extracellular matrix (ECM) [6]. However, current evidence suggests that these scaffolds typically play a passive role and do not actively contribute to bone regeneration [7]. Thus, there is an imperative to develop scaffolds that actively participate in the bone regeneration process.

Alginate (Sigma Aldrich, Gillingham, UK) is a natural polysaccharide which is derived from brown seaweeds. Also, it is industrially manufactured using various marine algae like *Laminaria hyperborea* and *Laminaria digitata* [8]. Chemically, alginate comprises a linear polymeric acid consisting of (1-4)-linked β-d-mannuronic acid (M) and α-l-guluronic acid (G) residues. When exposed to specific divalent cations, alginate can form stable scaffolds via ionic interactions between the cations and the carboxyl functional group of G units along the polymer chain [9]. It boasts high hydrophilicity, biocompatibility, relative cost-effectiveness, and widespread applications in the food and pharmaceutical industries. Cross-linked alginate is insoluble in aqueous solutions [10]. These biomaterials serve as three-dimensional (3D) scaffolds, with surfaces conducive to cellular adhesion, proliferation, and differentiation, fostering a conducive environment for tissue regeneration [11]. Alginate offers the advantage of biocompatibility and facile integration with drugs and cells, making it easily processable into 3D scaffolding material. However, pure alginate exhibits limitations such as weak mechanical and regenerative capabilities [12]. Hence, combining it with other biopolymers enhances its properties.

Carrageenan (Sigma Aldrich, Gillingham, UK), also derived from seaweed, is recognized for its ability to form gels. The gel properties it exhibits can vary depending on the type of carrageenan utilized, such as kappa, iota, or lambda carrageenan. Among these, kappa carrageenan stands out as the most suitable for applications in bone tissue engineering due to its gelation characteristics, mechanical strength, and structural resemblance to glycosaminoglycan (GAG) components like chondroitin-4-sulphate and dermatan sulphate. These properties make kappa carrageenan well-suited for use in tissue engineering [13]. GAGs are naturally occurring components of the extracellular matrix in the human bone and cartilage. The similarity in structure between carrageenan and GAGs is believed to enhance cell adhesion and proliferation [14]. Additionally, carrageenan exhibits both chondrogenic and osteogenic potential, owing to its abundant sulphate groups, which enable it to mimic the charged proteins found in the ECM [15].

These scaffolds offer both mechanical reinforcement and the ability to regulate the release of bioactive substances. When combined, alginate and carrageenan can produce composite scaffolds featuring enhanced mechanical strength and superior biological capabilities, thereby advancing the prospects for bone regeneration. Hence, this study aims at developing and evaluating alginate- and carrageenan-incorporated scaffolds for bone regeneration.

## Materials and methods

Study design

The study was an in vitro study carried out at Saveetha Dental College and Hospitals, Chennai, India. The Ethical Committee of the said institution accepted the investigation protocol (SRB/SDC/PERIO-2101/22/TH-130).

Scaffold fabrication**

**Figure 1:**
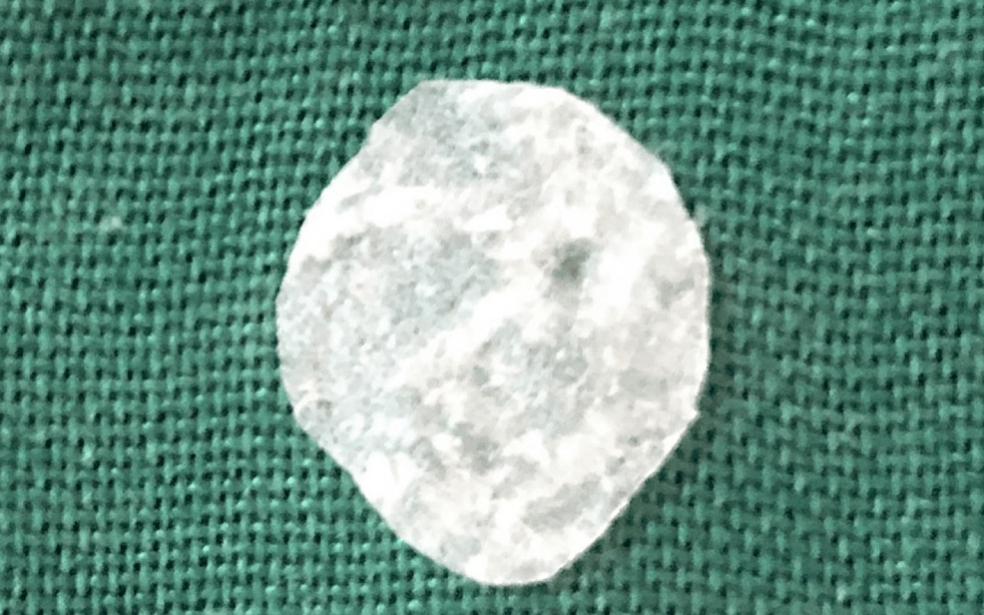
Alginate and carrageenan scaffold Figure 1 shows alginate- and carrageenan (2.5%)-incorporated scaffold prepared through the freeze-drying method

Two grams of alginate with a molecular weight ranging from 190 to 310 kDa was dissolved in distilled water and stirred until fully dissolved. Subsequently, it was combined with 2% kappa carrageenan, prepared by dissolving 2 grams of kappa carrageenan in 100 ml of distilled water. Similarly, concentrations of 2.5% of the alginate and carrageenan mixture were prepared. Divalent cations, specifically calcium chloride (CaCl2), were then added to the resulting solution. These divalent cations cross-link the alginate and carrageenan chains, inducing gel formation. The gelation process was initiated by gradually adding CaCl2 solution drop by drop to the mixture of alginate and carrageenan. To remove any residual, specimens were subjected to a series of ethanol washes for 15 minutes. Following this, the samples were kept in glycerol (Fisher Scientific, UK) and distilled water solution in a 1:10 ratio for 15 minutes. Subsequently, the samples were freeze-dried, and the resulting scaffolds were stored at room temperature (Figure 1).

Fourier transform infrared (FTIR) spectroscopy

The scaffold's functional group analysis was conducted using FTIR spectroscopy (Bruker Alpha II, Billerica, MA, USA). The samples were affixed to an attenuated total reflectance (ATR) crystal, and spectra were captured within the range of 500-3500 cm^-1^. Scans were performed across different areas of the sample to investigate the presence of various elements within it.

Tensile strength

The tensile strength was assessed using the universal testing machine (Instron Electroplus E3000, Norwood, MA, USA). Test samples measuring 10 × 15 mm were utilized for analysis at a crosshead speed of 10 mm/min. Upon fitting the membranes onto the analyzer, forces of 1.29 N, 1.26 N, and 1.25 N were applied to the test specimens and the control group (conventional collagen membrane, PerioCol®), respectively. The force at which the scaffold ruptured was recorded as the breaking force. Multiplying this breaking force by the area of the specimens yielded the tensile stress, and the deformation at the breaking force was considered the tensile strain.

Scanning electron microscopy (SEM) analysis

SEM analysis was done using a JEOL JSM-IT800 SEM to examine the structural morphology of the scaffold. The scaffolds were sectioned into small pieces using a razor blade, and samples were prepared. Platinum was sputtered onto the sections under vacuum, after which SEM analysis was carried out at a magnification of 500×.

Degradation test

The purpose of this test was to assess the degradation rate of the scaffold. Cut segments of the scaffold were immersed in 500 microliters of 1× phosphate-buffered saline (PBS) at 37°C. The initial dry weight (W0) was determined, followed by measuring the wet weight (Wt) at baseline and seventh, 14th, and 28th days. The degradation of the scaffold was directly linked to the rate of weight loss (% WL), calculated using the following formula: %WL = (W0 - Wt) / W0 × 100.

Statistical analysis 

Statistical analysis was done using IBM SPSS Statistics for Windows, V. 1.2 (IBM Corp., Armonk, NY, USA). The data was tested for mean and standard deviation. The statistical analysis for tensile strength was performed using the one-way ANOVA test to determine significant differences. Paired t-test was used for a degradation test to measure the statistical difference between groups, and a p-value of <0.05 was considered to be statistically significant.

## Results

FTIR spectroscopy

**Figure 2:**
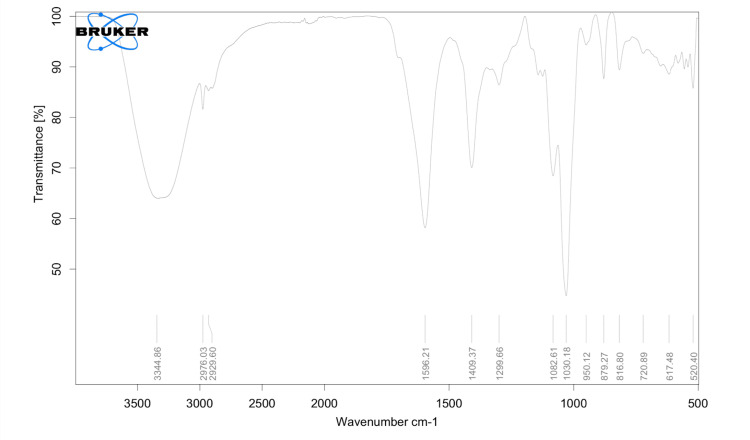
FTIR of alginate and carrageenan scaffold Figure 2 illustrates characteristic absorption peaks of alginate- and carrageenan-incorporated scaffold via spectroscopic analysis FTIR: Fourier transform infrared

The FTIR spectra obtained in the study helped identify the elemental composition of the materials present in the scaffold. Various peaks were observed, and the corresponding functional groups were identified. In the FTIR spectrum of alginate, absorption peaks were noted at 1596 cm^-1^ (indicating the C=O bond) and 1409 cm^-1^. Carrageenan displayed characteristic peaks associated with stretching vibrations of -OH and glycosidic linkage (C-O-C bond) at 3344 cm^-1^ and 1030 cm^-1^, respectively (Figure 2).

Tensile strength 

The tensile strength of the specimens was assessed using the universal testing machine from Instron. The observed tensile strain (displacement) for scaffold 1 and scaffold 2 was 371 ± 14 and 315 ± 17, respectively, at maximum forces of 1.29 N and 1.26 N, while for the control group, it was 308 ± 0.15 at a maximum force of 1.25 N. The tensile stress for scaffold 1 and scaffold 2 was determined to be 15.26 ± 0.05 MPa and 14.28 ± 0.06 MPa, respectively, whereas for the control group, it measured 14.26 ± 0.05 MPa (Table 1).

**Table 1:** Tensile strength analysis of scaffold 1, scaffold 2, and control group Table 1 represents the tensile stress and strain of alginate and carrageenan scaffold 1 and scaffold 2 of 2% and 2.5% concentration, respectively, and control group. The significant difference in the tensile stress was present (p = 0.03). Also, a significant difference was present in the tensile strain (p = 0.02). * represents a significant statistical difference

Specimen	Maximum force (N)	Tensile stress (MPa)	Tensile strain (displacement) at break (standard) (%)
Scaffold 1 (alginate + carrageenan: 2.5%)	1.29 ± 0.007	15.26 ± 0.05	371 ± 0.14
Scaffold 2 (alginate + carrageenan: 2%)	1.26 ± 0.005	14.28 ± 0.06	315 ± 0.17
Control group (PerioCol®)	1.25 ± 0.006	14.26 ± 0.05	308 ± 0.15
P-value		0.03*	0.02*

**Figure 3:**
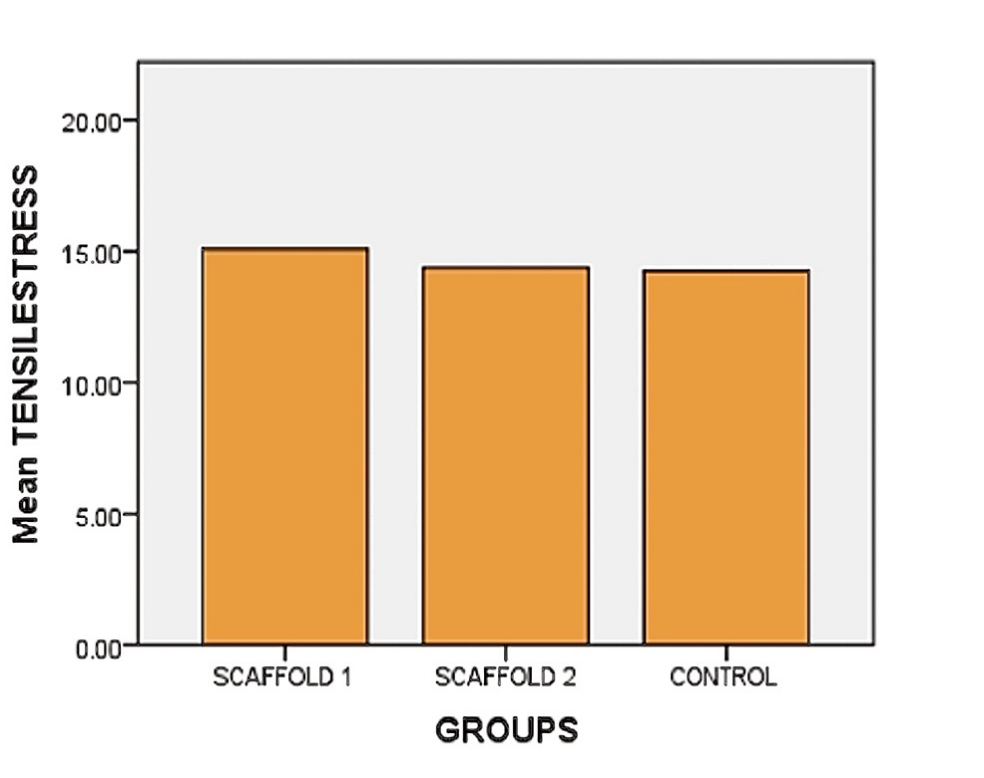
Tensile stress Figure 3 represents the tensile stress (MPa) of alginate and carrageenan scaffold 1 (alginate and carrageenan: 2.5%), scaffold 2 (alginate and carrageenan: 2%), and control group (PerioCol®; p = 0.03)

A notable difference in tensile stress was seen between scaffold 1 and scaffold 2 (p = 0.03) and also between scaffold 1 and the control group (p = 0.04). Graphs illustrating the tensile stress for all three groups are presented in Figure 3.

**Figure 4:**
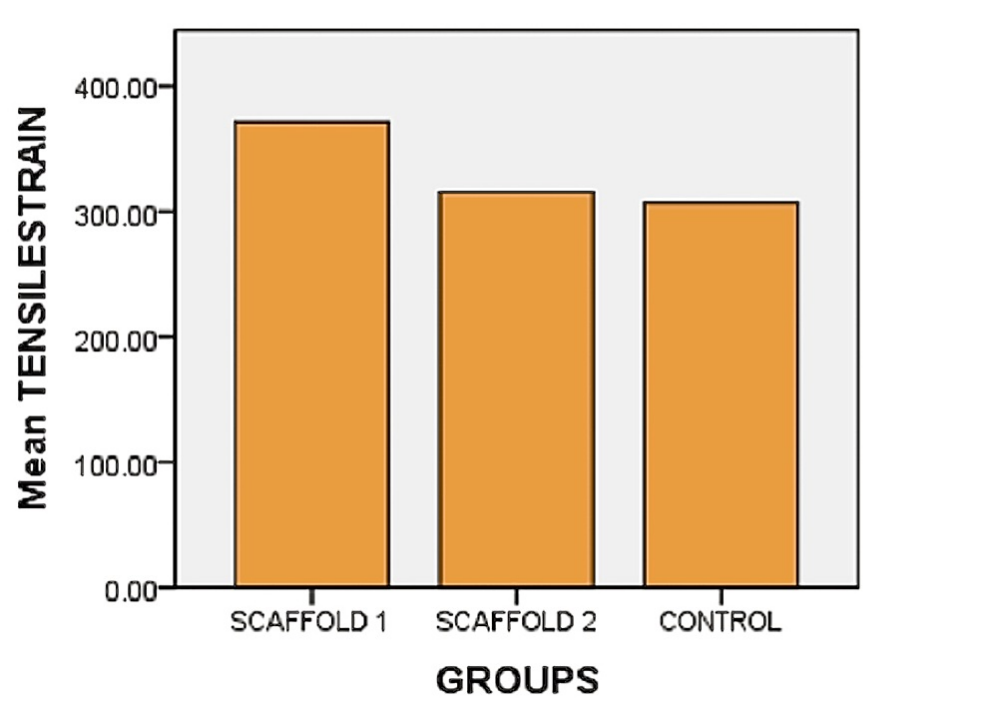
Tensile strain Figure 4 represents the tensile strain of alginate and carrageenan scaffold 1 (alginate and carrageenan: 2.5%), scaffold 2 (alginate and carrageenan: 2%), and control group (PerioCol®; p = 0.02)

Tensile strain also showed a significant difference between scaffold 1 and scaffold 2 (p = 0.03) and also between scaffold 1 and the control group (p = 0.04). Graphs illustrating the tensile strain for all three groups are presented in Figure 4. Considering that scaffold 1 exhibited superior tensile strength due to its higher concentration, further investigation was conducted using scaffold 1 as the test group.

SEM analysis

**Figure 5:**
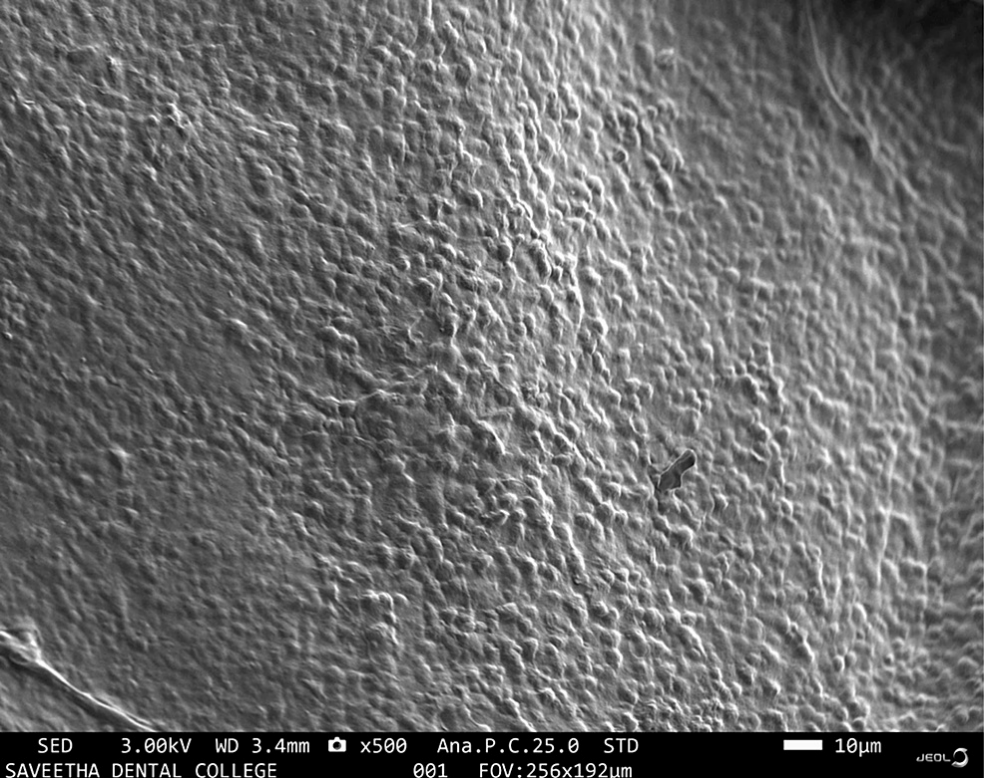
SEM analysis of alginate and carrageenan scaffold at 500× Figure 5 illustrates a SEM image showing small, regular, and homogenous pores at 500× magnification SEM: scanning electron microscopy

The SEM image acquired offered comprehensive insights into the surface characteristics, topography, and pore architecture of the alginate and carrageenan scaffold. The scaffold exhibited a sheet-like structure with discernible pores at 500× (Figure 5).

**Figure 6:**
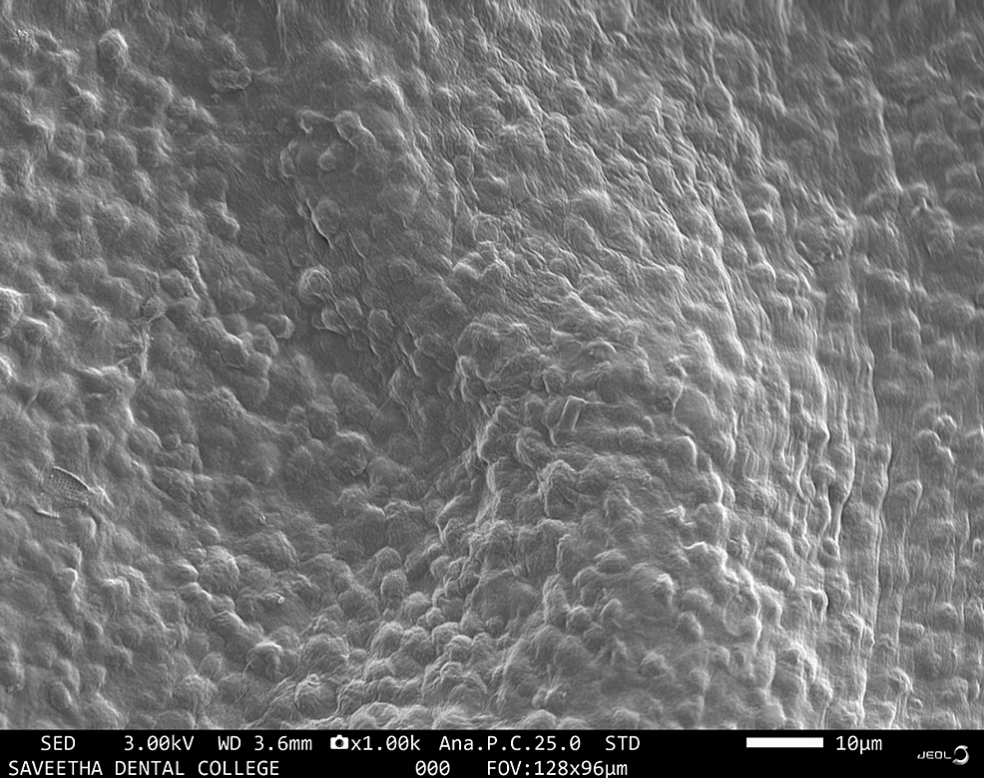
SEM analysis of alginate and carrageenan scaffold at 1000× Figure 6 illustrates a SEM image showing regular and homogenous pores at 1000× magnification SEM: scanning electron microscopy

The pore configuration within the scaffold appeared uniformly small and regular, featuring smooth walls and homogeneity at 1000× (Figure 6).

Degradation test

The changes in the weight of the scaffold were assessed in both groups on the first, seventh, 14th, and 28th days, respectively. Notably, significant weight variation in the control group was evident by the seventh day, with the fundamental structure of the fish collagen (PerioCol®) becoming increasingly fragile over time. In contrast, the test group membrane exhibited substantial weight variation by the 14th day, followed by a rapid decline in weight thereafter. Over the course of 28 days, the alginate and carrageenan scaffold maintained its fundamental structure. Conversely, the PerioCol® membrane demonstrated a swiffer degradation rate, with a loss of 13% of its baseline weight, whereas the alginate and carrageenan scaffold demonstrated a loss of less than 7% of its baseline weight (Table 2 ).

**Table 2:** Degradation analysis of the test group (alginate and carrageenan scaffold: 2.5%) and control group Table 2 illustrates the degradation rates as loss of scaffold weight (in %) of the test group and the control group at the first, seventh, 14th, and 28th days. Loss of weight % was seen higher for the control group than the test group on the 28th day

Immersion time period	Test group (alginate + carrageenan 2.5%) in milligrams	Loss of scaffold weight (in %)	Control group (PerioCol®) in milligrams	Loss of scaffold weight (in %)
1st day	48.3 ± 0.7	1	48.5 ± 0.3	1
7th day	42.5 ± 0.6	3	42.4 ± 1.5	5
14th day	40.4 ± 0.8	5	35.4 ± 0.5	8
28th day	38.5 ± 1.2	8	31.2 ± 1.6	12

**Figure 7:**
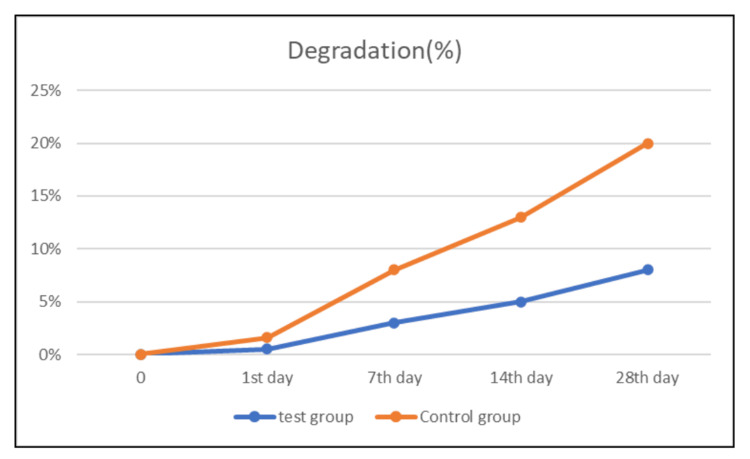
Degradation analysis for the test group and control group Figure 7 represents the degradation analysis for the test group (alginate and carrageenan scaffold: 2.5%) and control group

Graphical representation of the degradation analysis on the first, seventh, 14th, and 21st days showed a significant difference in the loss of weight (Figure 7).

## Discussion

Various synthetic and natural polymers are presently employed in scaffold synthesis for treating periodontal defects; nevertheless, the regenerative capacity of these scaffolds remains uncertain. Hence, there is considerable interest in advancing regenerative biomaterials to enhance outcomes and predictability [16]. Ongoing research is focused on exploring new materials for their regenerative potential and enhancing mechanical properties [17].

Thus, in the current investigation, a scaffold for bone regeneration was effectively created through the freeze-drying method using a blend of alginate and carrageenan. FTIR analysis of the resulting scaffold verified its elemental composition (C=O bond, -OH, and C-O-C bond), with characteristic absorption bands corresponding to alginate and carrageenan observed. These results align with previous research findings by Ramli et al. [18].

The SEM examination of the innovative scaffold revealed a uniform morphology with a consistent pore structure. The surface characteristics of scaffolds are known to influence various cellular behaviors including attachment, migration, proliferation, stem cell differentiation, and gene expression. SEM provides a 3D perspective of the sample surface, making it suitable for imaging scaffolds [19]. The pore size of the scaffold plays a crucial role in inhibiting excessive infiltration of fibrous tissue into the bone defect while allowing neoangiogenesis and osteogenesis [20]. Although the pore size was not quantified for the newly developed scaffold, the even appearance of the pores was observed at magnifications of 500× and 1000×. It can be inferred that this scaffold would facilitate the attachment, migration, and proliferation of the target cells. A previous study examining the microscopic appearance of a scaffold derived from marine polysaccharides presented similar images to those observed in this investigation [21].

Determining the mechanical properties of biomaterials is crucial for assessing their ability to be handled during surgical procedures and endure forces upon implantation at the defect site. In this investigation, the analysis of tensile strength (both tensile stress and strain) revealed notable differences in the test group with a higher concentration (alginate and carrageenan scaffold: 2.5%). Although membranes should possess flexibility and ease of cutting and shaping without sharp edges that avoid puncturing the tissues, meanwhile, it should be able to bear the masticatory load and remain at the site without getting distorted for the required amount of time and adapt closely to various defect morphologies [22]. A previous study reported that calcium alginate membranes displayed a comparable tensile strength of 14.54 ± 0.05 MPa [23]. Our observations suggest that this scaffold can endure biomechanical forces.

When alginate was cross-linked with carrageenan using Ca2+, molecular chains of alginate and carrageenan became entangled, resulting in a more stable cross-linked network that could endure for 28 days with only an 8% loss of initial weight. A suitable degradation rate is crucial for tissue-engineered scaffolds. Previous studies have shown that collagen-based membranes exhibit a high degradation rate, with a 15% loss of initial weight by the 28th day [24]. If degradation occurs too rapidly, the scaffolds may fail to adequately support the cells. Conversely, if the degradation rate is too slow, it could hinder the formation of new tissues [25]. In our investigation, the newly developed scaffold demonstrated enhanced stability, which is optimal for bone regeneration.

Limitations 

The full potential of GTR/guided bone regeneration (GBR) scaffolds can be thoroughly evaluated through human clinical trials. Consequently, numerous studies are needed to further assess these scaffolds in clinical settings. Concurrently, certain aspects of in vitro testing also require attention. The ability of scaffolds to transport and release growth factors should also be studied. Long-term degradation studies are necessary to evaluate scaffold stability in the oral environment. Moreover, the effects of temperature and pressure on degradation should be examined. The mechanical properties of scaffolds may vary within the oral cavity due to scaffold wettability, potentially altering tensile strength. Ultimately, the suitability of scaffolds for GTR/GBR procedures must be evaluated in clinical trials.

## Conclusions

The fabrication of the alginate and carrageenan scaffold using the freeze-drying technique was successful. Spectroscopic analysis (FTIR) validated the elemental composition of the newly developed scaffold, confirming the cross-linking of alginate and carrageenan. Physical and biological tests yielded satisfactory results. SEM analysis at high magnification revealed that the pore shape of the scaffold was uniform and consistent. Compared to PerioCol®, the tensile strength of the alginate and carrageenan scaffold increased with higher concentrations of both constituents. The novel scaffold demonstrated significant stability with an optimal degradation rate. The overall results of the study suggest that alginate and carrageenan scaffolds could be good candidates for bone regeneration; however, further work needs to be done in order to make them suitable for clinical use.
